# Prevalence of recurrent aphthous ulceration in Jordanian dental patients

**DOI:** 10.1186/1472-6831-9-31

**Published:** 2009-11-22

**Authors:** Rima Ahmad Safadi

**Affiliations:** 1 Department of Oral Surgery and Medicine, Faculty of Dentistry, Jordan University of Science and Technology, Irbid, Jordan

## Abstract

**Background:**

Reviewing the literature, no studies were cited to report the prevalence of recurrent aphthous ulceration in Jordan. The aim of this study is to report the prevalence of recurrent aphthous ulceration in Jordanian subjects.

**Methods:**

A total of 684 dental patients who attended Jordan University of Science and Technology interviewed and administered to fill questionnaires related to history, size, shape, and duration of recurrent aphthous ulceration. Other related questions were also asked.

**Results:**

About 78% of subjects experienced recurrent aphthous ulceration. Approximately 85% of ulcers were less than one cm in diameter, 66% were circular in shape, 92% were painful, 82% interfered with eating, and 55% located in lips and buccal mucosa. Only 50%of participants related ulcers to stress. Sixty eight percent reported no association with tiredness and 85% no association with types of food ingested. Of the 39% who had blood tests carried out, 7% had vitamin B12 and 4% hemoglobin deficiency.

**Conclusion:**

Recurrent aphthous ulceration is a common problem in Jordanian adults.

## Background

Recurrent aphthous ulceration was reported as the most common inflammatory ulcerative condition of the oral mucosa. [1,2] Aphthous ulcers are classified on the basis of ulcer size into major, minor or herpetiform. [3] Minor aphthous ulcers are small (less than one cm in diameter), well defined, shallow, and heal within two weeks without scars. Major ulcers, however, are bigger, deeper, and take up to six weeks to heal leaving a scar behind. Herpetiform ulceration is also characterized by small (3-6 mm), shallow ulcers which takes weeks to heal, but with many numerous ulcers at once. [3]

The etiology of recurrent aphthous ulceration is not understood. [4] No principal cause has been discovered, however attacks may be precipitated by, or associated with, local trauma, stress, food hypersensitivity, hormonal changes, microorganisms, and vitamin and trace element deficiencies. [5] Systemic conditions including genetic predisposition, immune dysregulation, and family history might play a role in recurrent aphthous ulceration in some patients. [6]

A diagnosis of recurrent aphthous ulceration depends mainly on history and clinical examination. Patients with mild recurrent aphthous ulceration usually do not require any treatment for the lesion. However topical cortocosteroids therapy may be used to reduce the frequency and severity of attacks. [7] The occurrence of aphthous ulcers appears to be less frequently in smokers than in non-smokers. [8,9] However, an increase experience of recurrent aphthous ulcerations was reported following smoking cessation. [10] Female had predominance in aphthous ulcers. [11,12] Aphthous ulcers are increased by increasing age and minor aphthous ulcers are 80% of suffered patient. [13]

The prevalence of recurrent aphthous ulceration was reported to be 40% in a sample of children of the United States. [14] Aphthous ulcers were reported to vary from 5 to 66% among different nations. [15,16] Reviewing the literature, no studies were cited to report the prevalence of recurrent aphthous ulceration in Jordan.

The aim of this study is to report the prevalence of recurrent aphthous ulceration in a sample of Jordanian subjects and to understand the independent factors that might be related to this condition.

## Methods

The sample of the present study comprised dental patients who attended Jordan University of Science and Technology's Dental Teaching Center for examination and treatment at a variety of dental disciplines. No attempt was made to exclude any subject for any reason. This study was approved by the Jordan University of Science and Technology (JUST) ethics committee through the Deanship of Scientific Research at JUST. Signed consent forms were obtained from all participants before conducting the study.

Subjects were interviewed and administered and asked to fill a questionnaire specially prepared for this project. The questionnaire includes questions related to the socio-demographics of the subjects. It also includes questions related to history, size, shape and duration of recurrent aphthous ulceration. Other questions were related to management of ulceration and also related to other independent factors that might be related to the condition.

Collected data were recorded on paper forms, and were then entered, verified, processed and analyzed using a software package SPSS program version 12 (SPSS Inc; Chicago, USA 2003). The frequency distribution of recurrent aphthous ulceration was calculated. Chi-square test was also used to compare the prevalence of recurrent aphthous ulceration between categories of age, gender, education, occupation and family history of recurrent aphthous ulceration.

## Results

The number of subjects invited to participate were 800 subjects. Subjects who accepted participation in the interview were 684 participants. About 45% of participants were males and 55% were females. The mean age of participants was 37.5 years and ranged from 13 years to 68 years old. The mean years of education were 12.7 years and ranged from 0 to 18 years. From those who were employed, 47% were engaged in professional occupation and 53% were non-professional workers. About 31% of participants were smokers and male smokers were found to be more prevalent (39%) than female smokers (24%).

Table 1 presents information related to recurrent aphthous ulceration experience among participants. Approximately 78% of participants reported that they experienced recurrent aphthous ulceration in the past. About 85% of the reported ulcerations were less than one cm in diameter, and about two-thirds of them were circular in shape. Approximately half of participants reported that ulcerations were single ulcerations while the other half reported them as multiple ulcerations. About 92% subjects reported that the ulcers were painful and 82% claimed that the ulcerations interfered with food eating and swallowing. Lips and buccal mucosae were the commonest sites of ulcerations (55%), whereas gingivae or tongues were affected in one-fifth of subjects. Floor of the mouth was the least affected site by recurrent aphthous ulceration (8%). The duration of ulceration was reported to be less than a week for about two-thirds of participants while about 29% of participants reported that the ulceration extended up to two weeks and only minority (4%) reported that the duration of ulceration extended longer than two weeks. Regarding family history of recurrent aphthous ulceration, 66% of subjects reported that other family member suffered previously from recurrent aphthous ulcerations. Itching prior to ulceration was experienced only by 38% of subjects.

**Table 1:** Information related to recurrent aphthous ulceration experience among participants

Variable	Category	No.	%
History of aphthous ulcer	Yes	534	78.1
	
	No	150	21.9

Size of the ulcers	>1 cm	76	15.4
	
	< = 1 cm	418	84.6

Shape of the ulcer	Circular	330	65.7
	
	Non-circular	172	34.3

Number of ulcers	Single ulcer	254	49.2
	
	Multiple ulcers	262	50.8

Are the ulcers painful	Yes	482	91.6
	
	No	44	8.4

Interfere with eating or swallowing	Yes	408	81.9
	
	No	90	18.1
	
	Lip and inner cheek	288	55.0

site of the ulcers	Floor of the mouth	42	8.0
	
	Gingiva	96	18.3
	
	Tongue	98	18.7
	
	< 7 days	336	66.9

Duration of the ulcers	7-14 days	146	29.1
	
	> 14 days	20	4.0

Itching prior ulceration	Yes	190	37.5
	
	No	316	62.5

Any other family member suffering from recurrent aphthous ulcerations	Yes	408	66.4
	
	No	206	33.6

Table 2 presents independent factors that might predispose to the condition with their management among participants. Female participants reported that they had recurrent aphthous ulceration (82%) more than males (73%) and the difference between the genders was statistically significant (p = 0.003). Approximately 51% of participants reported that their recurrent aphthous ulcerations were related to stress; however, about 68% did not relate the ulceration to tiredness. Furthermore, 85% of participants did not relate the condition to eating certain types of food. Regarding blood test results, about 39% of participants agreed to have a blood test as part of an investigation of the cause of aphthous ulcer. From those who underwent a blood test, 7% and 4% reported that they had decreased B12 vitamin and hemoglobin, respectively.

**Table 2:** Related independent factors and management of recurrent aphthous ulceration among participants

Variable	Category	No.	%
Gender with history of ulceration	Male	226	72.9
	
	Female	308	82.4

Related to stress	Yes	188	51.1
	
	No	180	48.9

Related to tiredness	Yes	119	32.3
	
	No	249	67.7

Associate with certain types of food	Yes	76	14.6
	
	No	444	85.4

Blood testing for aphthous	Blood not tested	414	60.5
	
	Blood tested normal	194	28.4
	
	Decreased B12	50	7.3
	
	Decreased hemoglobin	26	3.8

Do you think treatment of aphthous effective	Yes	174	59.2
	
	No	120	40.8

Type of treatment used for aphthous ulcers	Live with it without treatment	322	62.9
	
	Use of pain killer and antibiotics without dental consultation	100	19.5
	
	Consult dentists	60	11.7
	
	Use of steroids/cortisones	30	5.9

Reason for not consulting dentists for aphthous	It is a simple known problem	304	67.3
	
	Recurrent aphthous ulceration is not dental problem	76	16.8
	
	Dental treatment is not effective	72	15.9

Regarding treatment of recurrent aphthous ulceration, about 41% of participants thought that the treatment of recurrent aphthous ulceration was not effective. When participants were asked how they dealt with recurrent aphthous ulceration, 63% reported the use of no treatment and just to live with it. However, about 20% used painkillers and antibiotic medications without medical consultation. A small percentage of participants used steroids (6%) and about only 12% consulted dentists for the ulceration. Those who did not consult dentists, 67% thought that aphthous is a simple problem and did not required dental consultation. Approximately 17% thought recurrent aphthous ulceration is not a dental problem and 16% thought that treatment for recurrent aphthous ulcerations is not working and they were tired from visiting dentists.

## Discussion

Recurrent aphthous ulceration is quite common condition affecting the oral cavity, yet it is an important condition since it can be distressing and cause suffering and pain. In addition, it interferes with normal life activities by affecting eating and swallowing of sufferers. Studying the prevalence of recurrent aphthous ulceration is important since it gives insight into the proportion of people who suffer from the condition as well as the possible causal factors.

Jordan University of Science and Technology provide free-of-charge dental examination and treatment to all patients regardless of their dental insurance. This service is part of dental student training on most of dental disciplines. The results of this study reflect the prevalence of recurrent aphthous ulceration only in patients attended dental clinics at JUST. However, there is no reason to believe that this group of patients is different from other residents in north Jordan.

Subjects were asked, "Have you ever suffered recurrent aphthous ulceration in your life?". This type of studies depends mainly on patients' recollection of the condition in the past. Few patients were considered not having aphthous ulcers because they could no recall if they had this condition in their life, therefore recurrent aphthous ulceration occurrence for those patients might be unrealistic.

Comparison of the present results with those from previous studies should be undertaken with caution due to differences in the study design, sample size, and geographical location. The result of this study was higher than that reported from USA, [17] and many other countries. [18] This could be explained by that Jordanian subjects might be different than other populations in term of genetic predisposition, stress, lifestyle and other related factors.

Reviewing the literature, approximately 80% of patients with recurrent aphthous ulcerations were exhibiting minor type. [18] This is consistent with the results of the present study. Jordanian females were found to have more ulceration than males. These results are also in line with other studies. [11,17]

This study is the first to describe the prevalence and distribution of recurrent aphthous ulceration among Jordanian residents. No other publication was found to address the prevalence of this condition among Jordanian adults.

## Conclusion

Understanding the prevalence and distribution of recurrent aphthous ulceration among Jordanian population will give an indication about the proportion of people who suffer the condition and who need dental management. Knowledge about the increased proportion of Jordanian people with recurrent aphthous ulceration might help dental practitioner in reaching the proper diagnosis of the ulcers affecting oral cavities and in providing information to patient to enhance their awareness about the condition.

## Competing interests

There are no competing interests (political, personal, religious, ideological, academic, intellectual, commercial or any other) to declare in relation to this manuscript.

## Authors' contributions

All work is the solely work of the author of this study.

## Pre-publication history

The pre-publication history for this paper can be accessed here:

http://www.biomedcentral.com/1472-6831/9/31/prepub
